# Reassortments among Avian Influenza A(H5N1) Viruses Circulating in Indonesia, 2015–2016

**DOI:** 10.3201/eid2503.180167

**Published:** 2019-03

**Authors:** Desniwaty Karo-karo, Rogier Bodewes, Hendra Wibawa, Made Artika, Eko Sugeng Pribadi, D. Diyantoro, Widya Pratomo, Agus Sugama, Nani Hendrayani, Iin Indasari, Michael Haryadi Wibowo, David Handojo Muljono, Jan Arend Stegeman, Guus Koch

**Affiliations:** Utrecht University, Utrecht, the Netherlands (D. Karo-karo, R. Bodewes, J.A. Stegeman);; Indonesian Ministry of Agriculture, Jakarta, Indonesia (D. Karo-karo, H. Wibawa);; Bogor Agricultural University, Bogor, Indonesia (I.M. Artika, E.S. Pribadi, D. Diyantoro, W. Pratomo);; Eijkman Institute for Molecular Biology, Jakarta (I.M. Artika, D.H. Muljono);; Livestock and Animal Health Agency of District Subang, Subang, Indonesia (A. Sugama);; West Java Province Animal Health Laboratory, Cikole, Indonesia (N. Hendrayani);; West Java Province Animal Health Agency, Bandung, Indonesia (I. Indasari);; Gajah Mada University, Yogyakarta, Indonesia (M.H. Wibowo);; Wageningen Bioveterinary Research, Lelystad, the Netherlands (G. Koch)

**Keywords:** HPAI, reassortment, phylogenetic analysis, viruses, influenza, West Java, Indonesia, respiratory infections, highly pathogenic avian influenza

## Abstract

Highly pathogenic avian influenza (HPAI) A(H5N1) viruses have been circulating since 2003 in Indonesia, with major impacts on poultry health, severe economic losses, and 168 fatal laboratory-confirmed human cases. We performed phylogenetic analysis on 39 full-genome H5N1 virus samples collected during outbreaks among poultry in 2015–2016 in West Java and compared them with recently published sequences from Indonesia. Phylogenetic analysis revealed that the hemagglutinin gene of all samples belonged to 2 genetic groups in clade 2.3.2.1c. We also observed these groups for the neuraminidase, nucleoprotein, polymerase, and polymerase basic 1 genes. Matrix, nonstructural protein, and polymerase basic 2 genes of some HPAI were most closely related to clade 2.1.3 instead of clade 2.3.2.1c, and a polymerase basic 2 gene was most closely related to Eurasian low pathogenicity avian influenza. Our results detected a total of 13 reassortment types among HPAI in Indonesia, mostly in backyard chickens in Indramayu.

Highly pathogenic avian influenza viruses (HPAI) continue to be a major global problem for both animal and human health. Since the first outbreak of HPAI A(H5N1) in Guangdong, China, in 1996, these viruses have caused outbreaks in various species of birds globally. HPAI H5N1 is endemic in multiple countries and causes a major impact on poultry health and severe economic losses. In addition, >860 laboratory-confirmed human cases of HPAI H5N1 have been reported to the World Health Organization (WHO). In Indonesia, 200 laboratory-confirmed human cases of avian influenza A(H5N1) have been reported, with a case-fatality rate of 84%, which is higher than the current global case-fatality rate of 53% (*1*). The zoonotic potential of HPAI is a global public health concern, particularly in preventing a potential pandemic (*2*,*3*).

In Indonesia, HPAI H5N1 has been endemic in poultry since 2003 and continues to cause major economic losses to both poultry industry and backyard farms. The disease has been reported in 32/34 provinces, resulting in the death of millions of birds (*4*,*5*) and the closure of many farms in high-incidence areas (*6*). While HPAI H5N1 viruses continuously circulated among poultry in Indonesia during 2003–2010, the hemagglutinin (HA) genes evolved from clade 2.1 into multiple subclades, according to the unified nomenclature system for the HA gene of HPAI H5N1 virus (*7*). In 2012, a new virus classified as clade 2.3.2.1 was detected in ducks, suggesting a new incursion of HPAI H5N1 viruses in Indonesia from other parts of Southeast Asia (*7*–*9*). Vaccination programs have been applied to control the spread of HPAI H5N1 but have not prevented it because of low vaccination coverage and use of unlicensed vaccines. These problems have led to the emergence of antigenically distinct HPAI H5N1 virus clades in Indonesia (*10*). In addition to the continuous circulation of HPAI H5N1 viruses in poultry, transmission to humans has been reported in Indonesia since 2005 (*1*).

Clarifying the epidemiology of HPAI H5N1 requires more intense monitoring of outbreaks of HPAI in Indonesia and performing genetic and phylogenetic analysis on viruses detected during these outbreaks. However, recent information on the genetic divergence of HA, and in particular on other gene segments, is very limited (*8*,*11*–*13*), and samples are often collected in a nonsystematic way. Therefore, the aim of this study was to perform genetic and phylogenetic analysis on recent HPAI H5N1 viruses that were obtained from poultry during active searches for outbreaks in West Java, a province of Indonesia. West Java was selected for this study because it has a high poultry density, multiple different farming systems and live-bird markets, and several environmental components that all form risk factors for HPAI H5N1 virus transmission. Moreover, because a high percentage of the land in this region is paddy fields and water sources, free-ranging ducks and chickens undermine the effectiveness of prevention and control measures, resulting in the continuous circulation of the virus (*14*,*15*).

## Materials and Methods

### Sample Collection

**Figure 1:**
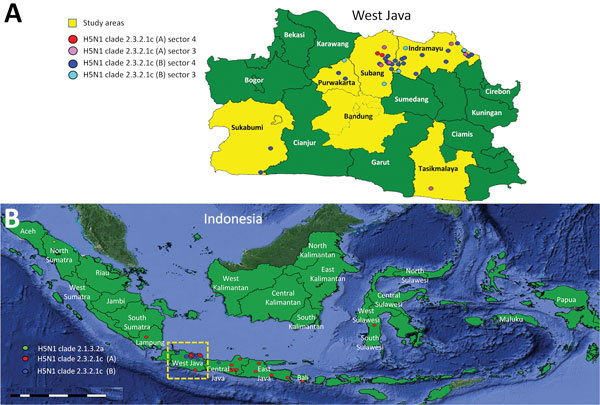
Locations of sampling areas and of different hemagglutinin (HA) clades in study of avian influenza A(H5N1) viruses circulating in Indonesia, 2015–2016. A) West Java Province; B) location of province in Indonesia (box). Data were compiled from this study and additional sequence data of Directorate General for Livestock Services, the Indonesian Ministry of Agriculture, and submitted to GenBank (accession nos. EPI1009273–463).

During April 2015–October 2016, district animal health officers of the West Java Animal Health Authority collected samples from birds in 6 districts of West Java Province: Subang, Indramayu, Tasikmalaya, Purwakarta, Sukabumi, and Bandung (Figure 1). The districts were chosen on the basis of the history and recurrence of HPAI outbreaks. In addition, these districts have multiple sectors of poultry farms using various production systems and a high density of poultry farms that have >50 birds/farm (*4*,*16*).

The samples were collected after detection of clinical signs in or increased mortality of birds. The criteria for increased mortality was set at >5% of the population in birds vaccinated against H5N1 and 10% in those unvaccinated for 2 consecutive days. When the criteria were met, oropharyngeal and cloacal samples were collected from 5 sick birds and pooled into viral transport medium containing brain–heart infusion broth and antimicrobial drugs according to European Union instructions (http://extwprlegs1.fao.org/docs/pdf/eur65757.pdf). The specimens were kept chilled and shipped by overnight courier to the 2 collaborating veterinary laboratories, Disease Investigation Center (DIC) Subang and West Java Animal Health Laboratory Cikole.

### Sample Screening

We tested the collected samples in veterinary laboratories using a national protocol for influenza A screening developed from a real-time reverse transcription PCR (RT-PCR) targeting the matrix gene. Specimens with a cycle threshold value <30 were inactivated using binding buffer of High Pure Viral RNA kit (Roche Applied Science, http://www.roche.com), and transported to the Eijkman Institute for Molecular Biology in Jakarta for Sanger sequencing. Two additional HPAI H5N1–positive samples, collected in 2016 and obtained from the Animal Health Laboratory (AHL) Cikole of West Java, were also inactivated and included in this study for Sanger sequencing.

### Sequencing

At the Eijkman Institute, we rescreened the specimens and extracted RNA in accordance with the protocol of the manufacturer and synthesized cDNA by Invitrogen Super Script III First-Strand Synthesis SuperMix (Thermo Fisher Scientific, http://www.thermofisher.com) with Uni12 primer (*17*). On specimens that tested positive in this PCR, we performed additional PCRs to amplify other gene segments present in the samples. We performed amplification of the full genomes of HPAI H5N1 viruses using a 2-step RT-PCR TaKaRa Z-Taq DNA Polymerase (Takara Bio, http://www.takarabio.com) or Toyobo KOD FX Neo (Toyobo, http://www.toyobo-global.com) if the genomes were not successfully amplified using the Takara product.

The primers used were primarily designed by Wageningen Bioveterinary Research. We obtained additional primer sequences from the Australian Animal Health Laboratory and from scientific literature (*17*,*18*) and applied them to unsuccessfully sequenced gene fragments that could not be amplified by standard primers. We purified the amplified PCR products with Roche High Pure PCR Product Purification Kit (Roche) or Zymoclean Gel DNA Recovery Kit (Zymo Research, https://www.zymoresearch.com) for PCR products for which gel separation was necessary, and subsequently sequenced them using a BigDye Terminator v3.1 Cycle Sequencing Kit in an ABI 3130 Genetic Analyzer (both from Thermo Fisher).

### Genetic and Phylogenetic Analysis

**Figure 2:**
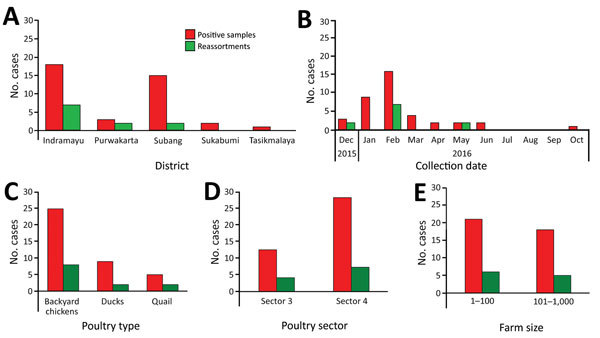
Number of samples in study of avian influenza A(H5N1) viruses circulating in Indonesia, 2015–2016, by district (A), time (B), poultry type (C), poultry sector (D), and farm size (E) from which the complete HPAIV H5N1 genome could be obtained.

We assembled and edited sequences with Lasergene SeqMan Pro version 12 (DNASTAR, http://www.dnastar.com) and aligned them by using MUSCLE (*19*). We initially determined HA clade of sequenced HPAI H5N1 viruses using the Highly Pathogenic H5N1 Clade Classification Tool of the Influenza Research Database (https://www.fludb.org) and confirmed results through further phylogenetic analysis (*20*). We estimated phylogenetic inference using the maximum-likelihood method with 1,000 bootstrap replicates (Figure 2; Appendix 1 Figure 1). We chose the most suitable substitution rates and pattern model based on the lowest Akaike information criterion for each alignment. Evolutionary distances were computed using average pairwise distance (APD) between and within sequence groups. Evolutionary analyses and APD were conducted in MEGA6 (*21*).

**Figure 3:**
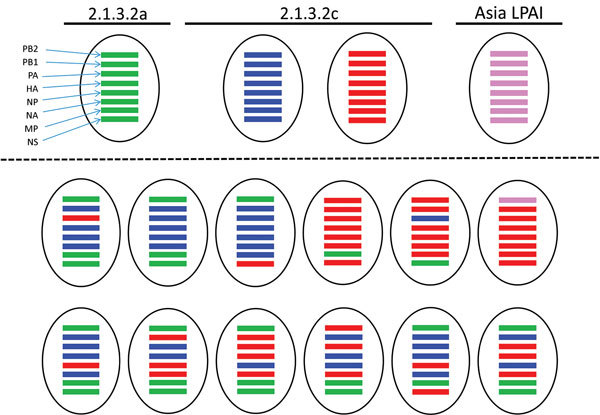
Reassortment events of avian influenza A(H5N1) viruses in samples from Indonesia, 2015–2016, some of which were confirmed using maximum-likelihood analysis with parent strains clade 2.3.2.1c, 2005-11 (clade 2.1.3.2a), and Asia low pathogenicity avian influenza virus. Parent strains appear above the dotted line and 13 detected reassortment types below the dotted line. HA, hemagglutinin; LPAI, low pathogenicity avian influenza virus; MP, matrix protein; NA, neuraminidase; NP, nucleoprotein; NS, nonstructural protein; PA, polymerase; PB1, polymerase basic 1; PB2; polymerase basic 2. Green bars indicate clade 2.1.3.1a; blue, clade 2.1.3.2c subgroup A; red, clade 2.1.3.2c subgroup B; violet, Asia LPAI.

We aligned the sequences of HPAI H5N1 gene segments collected during this study with reference sequences from GenBank (Appendix 1 Figures 1–8) using BLAST (https://blast.ncbi.nlm.nih.gov/Blast.cgi). We included in the analysis sequences obtained from viruses detected during other recent outbreaks in Indonesia (2014–2016). These viruses had been collected via passive outbreak surveillance by the Disease Investigation Centres (DIC) in Medan, Sumatra; Wates, Central Java; and Denpasar, Bali, under the Directorate General for Livestock and Animal Health Services and the Indonesian Ministry of Agriculture (DGLAHS-MoA). Viruses were submitted by DIC Wates of DGLAHS-MoA to GenBank, and then downloaded to GISAID (https://www.gisaid.org; accession nos. EPI1009273–463) (Appendix 2 Table 1). For sequencing, we used mainly viruses from original material, as well as some isolates obtained after 1–2 passages in embryonated chicken eggs. We deduced reassortment events on the basis of deviant location of sequences in maximum-likelihood trees of different gene segments.

We used deduced HA amino acid sequences to calculate estimated antigenic distances of viruses based on 27 aa residues in HA, as described previously (*22*). We measured the antigenic distances with 3 HPAI H5N1 strains that are or were routinely used to vaccinate poultry in Indonesia: A/chicken/Legok/2003 (clade 2.1.1); A/chicken/West Java/PWT-WIJ/2006 (clade 2.1.3.2); and A/duck/Sukoharjo/BBVW-1428–9/2012 (clade 2.3.2.1c). We used a *t*-test to estimate the significance of the comparison between the 2 averages of antigenic distances.

## Results

### Detection and Sequencing of HPAI Viruses

A total of 76 pooled samples were collected from various districts of West Java, Indonesia (Figure 1). We observed the highest number of outbreaks in Indramayu in February 2016. During April 2015–October 2016, a total of 56 of the samples tested positive for influenza A virus by real-time RT-PCR with a cycle threshold value <30. We obtained the complete genome from 37 oropharyngeal samples and 2 swab specimens of the 55 samples and used these sequences in the analysis. Positive samples with complete genomes were mostly collected in Indramayu (46.15%, 95% CI 30.5%–61.8%) and Subang (38.46%, 95% CI 23.2%–53.7%); the highest peak came in February 2016 (41.3%, 95% CI 25.6%–56.5%), and most positive samples came from backyard chickens (69.23%, 95% CI 54.74%–83.71%). The positive samples were primarily from sector 4 (69.23%, 95% CI 52.4%–83%), from farms with <100 birds/farm (53.85%, 95% CI 37.2%–70%) (Figure 2). Sequences comprising the whole genome were submitted to GISAID (Appendix 2 Table 1).

### Phylogenetic Analysis of HPAI H5N1 Genes

Analysis of obtained hemagglutinin (HA) and neuraminidase (NA) nucleotide and deduced amino acid sequence data confirmed that viruses in our samples were HPAI H5N1 with polybasic cleavage motif (Q-R-E-R-R-R-K-R-G-L-F) and (Q-R-E-K-R-R-K-R-G-L-F). Phylogenetic analysis showed that the HA genes of the HPAI H5N1 viruses in our study samples all belong to clade 2.3.2.1c. In-depth analysis revealed that Indonesia 2015–2016 HPAI H5N1 clade 2.3.2.1c has evolved into 2 putative new subgroups, A and B. The APD between the 2 subgroups within clade 2.3.2.1c was >1.5% (3.3% ± 0.4%); the bootstrap value was >60%; and the APDs within the 2 groups within clade 2.3.2.1c were <1.5% (0.9% ± 0.1% for subgroup A and 1.9% ± 0.2% for subgroup B). One sample collected by DIC Medan in 2016 from Sumatra Island was identified as clade 2.1.3.2a (Appendix 1 Figure 1).

We observed the evolution of clade 2.3.2.1c of Indonesia 2015–2016 HPAI H5N1 viruses into putative new subgroups (A and B) for the polymerase basic 1 (PB1), polymerase (PA), nucleoprotein (NP), and neuraminidase (NA) genes, as became apparent from comparing respective phylogenetic trees of these genes (Appendix 1 Figures 2–5). The APDs of the PB1, PA, NP, and NA genes were computed, although APD for these genes has not been used yet for HPAI nomenclature. The APD between the 2 different subgroups A and B within clade 2.3.2.1c viruses was 2.3% ±0.3% for PB1, 2.4% ±0.3% for PA, 2.1% ±0.3% for NP, and 3.4% ±0.3% for NA; and the APDs within the 2 different subgroups of clade 2.3.2.1c were 0.8% ±0.1% (A) and 1.6% ±0.2% (B) for PB1, 0.7% ±0.1% (A) and 1.3% ±0.1% (B) for PA, 0.6% ±0.1% (A) and 1.1% ±0.1% (B) for NP, and 0.7% ±0.1% (A) and 1.9% ±0.2% (B) for NA.

We identified 4 different variants of PB2 in HPAI H5N1 cases from Indonesia in 2015–2016, whereas MP and NS consisted of 3 variants. One of the 4 variants in the PB2 gene of HPAI H5N1 viruses collected by DIC from poultry outbreaks in Central and East Java in 2016 was similar to PB2 of LPAI from Asia (Appendix 1 Figures 1, 7, and 8).

### Detection of Possible Reassortments

Analysis of obtained sequence data by the maximum-likelihood method revealed the presence of multiple reassortments of HPAI H5N1 virus gene segments of different viruses circulating in Indonesia, using viruses of clade 2.3.2.1c, 2.1.3.2a, and Asia LPAI as parent strains (Figure 3). Based on the complete genome sequences of 37 positive samples, we identified the district with the most reassorted viruses as Indramayu (20.5%, CI 95% 9.3%–36.5%). The month with the largest proportion of infections was February 2016 (18%, 95% CI 7.5%–33.5%), and the type of poultry with the largest proportion of infections was backyard chickens (15.4%, 95% CI 5.9%–30.5%). We identified ≈18% (95% CI 7.5%–33.5%) of reassorted viruses in poultry sector 4; 15.4% (95% CI 5.95%–30.5%) were in farms with <100 birds/farm (Figure 3).

### Antigenic Distance Based on Genetic Distance

It has been demonstrated recently that genetic distances in 27 selected amino acid residues of the HA of HPAI H5 viruses correlate with antigenic distances (*22*). These 27 positions correlate closely with antigenicity and are close to receptor binding sites (*23*,*24*). We observed amino acid changes in the HA of the HPAI H5N1 viruses analyzed in our study at 19/27 selected residues: N72D, D97N, Q115H, S129L, S133A, P136S/P136L, L138Q, S140N, P141S, N154D/N154S, R162K, S163G/S163N/S163T, D183N, E184G, A185G, T188I, K189R/K189M, R212K, M226I (Appendix 2 Table 4).

Results show that the estimated average antigenic distance of HPAI H5N1 viruses from subgroup A was slightly smaller than from subgroup B to the most recent seed virus vaccine, A/duck/Sukoharjo/BBVW-1428-9/2012. Not surprisingly, these average antigenic distances were lower than to older seed vaccine strains of different clades (A/chicken/Legok/2003, A/chicken/West clade 2.1, and Java/PWT-WIJ/2006 clade 2.1.3.2). In all cases, the distance difference between subgroup A or B and the 3 seed vaccine strains was significant (p<0.05) (Appendix 2 Tables 4, 5).

## Discussion

We performed genetic and phylogenetic analysis on 39 complete genomes of HPAI H5N1 viruses obtained from recent outbreaks in West Java, Indonesia. The results of genetic analyses of the samples indicated that H5N1 clade 2.3.2.1c viruses are currently circulating predominantly in West Java and Sumatra. The finding of a single clade 2.1.3.2a virus, however, showed that this clade is still present in Indonesia. More systemic surveillance is required to confirm the prevalence of HA clade 2.1.3.2a viruses in Sumatra and Java. Of interest, we detected 2 new subgroups HA within clade 2.3.2.1c. These subgroups are candidate subclades; they share a common node, monophyletic grouping with bootstraps values >60, and APD between groups of >1.5% and within groups of <1.5%, fulfilling the criteria designed by the World Health Organization/World Organisation for Animal Health/Food and Agriculture Organization (WHO/OIE/FAO) H5 Evolution Working Group (*7*).

The diversity we detected in the HA subgroups of HPAI viruses in Indonesia in 2015–2016 we also detected in gene segments PB1, PA, NP, and NA, as was apparent by determination of the APD. However, although the APD between the groups was >2%, not all bootstrap values were >60. At the least, the calculated APD of PB1, PA, NP, and NA suggest that genetic variation of these genes is similar to that in HA.

The antigenic distances we deduced of the differences of 27 aa that determine antigenicity vaccination effectiveness of West_Java/PWT-Wij/2006 vaccines are expected to be lower against clade 2.3.2.1c than against clade 2.1.3.2a. Whether immunity induced by routine vaccination practices actually did facilitate (*25*–*28*) the replacement of 2.1.3.2a viruses by clade 2.3.2.1c after its incursion in Indonesia in 2012 needs further investigation. Whether vaccination also played a role in the emergence of subgroup B viruses is less likely; the difference in the antigenic distance between subgroup B and the vaccine virus A/duck/Sukoharjo/BBVW-128-9/2012, which came into use after 2012, is rather small and only just significant. Additional studies of other variables that might have affected the evolution of H5N1 virus in Indonesia, such as transmission efficiency of the viruses in different hosts, are required to prove or reject a possible role of vaccination. In all cases, the observed genetic variation combined with its effect on antigenicity illustrates the need for continued intense surveillance and prompt genetic characterization. Calculating antigenic distances based on the 27 aa of HA could greatly speed up the process of seed virus selection because serologic analyses, antigenic cartograph and experimental vaccination-challenge experiments are time-consuming and costly processes. However, such studies are still crucial to confirm the validity and reliability of this antigenic distance method for seed selection.

We observed the evolution of clade 2.3.2.1c into 2 subgroups in 2 different locations. One subgroup within this clade (A) was observed mostly in West Java, whereas another subgroup (B) was seen in diverse regions of Indonesia (Figure 1; Appendix 1). Additional studies are needed to confirm that there are indeed geographic differences between subgroups A and B and to elucidate possible causes, such as differences in vaccination strategies and differences in trade connections (*29*).

We identified reassortment events in West Java, mostly in backyard chickens in Indramayu. The high poultry density, the presence of different poultry types, and the frequent contacts between poultry farms and between domestic poultry and wild birds may have led to reassortment in West Java (*14*). A parallel study on contacts of different poultry sectors revealed that backyard chicken farms have the highest contact rate (*30*), which may have facilitated reassortment in West Java. Of interest, a recent study described reassortant HPAI H5N1 viruses in samples collected from live-bird markets associated with suspected human HPAI H5N1 cases in Indonesia (*13*). More intense surveillance programs are required to confirm the prevalence and distribution of the clade 2.1.3.2a and 2.3.2.1c subgroups and its reassortments and to be able to unveil the transmission of HPAI from different sectors, vaccination practices, and regions.

Reassortments between influenza viruses can only occur when a host cell is infected by >2 viruses with discrete genomes and when mixing within the host cells produces a hybrid genotype from segments of different parental strains. Because such events are dependent on simultaneous infections with multiple viruses, reassortments are more likely to occur at hotspots such as live-bird markets where different types of birds originating from many different farms, and potentially infected with different viruses, come together (*29*,*31*). Some computational methods have recently been developed to identify a putative reassortment event (*32*,*33*). In this study, the events were identified by maximum-likelihood phylogeny and genetic distance-based methods; we reconfirmed selected reassortments by Graph Incompatibility based on Reassortment Finder using Markov chain Monte Carlo computational methods (data not shown).

Phylogenetic analysis of PB2, M, and NS indicated reassortment between viruses circulating in Indonesia. The detection of 3 different variants of M and NS, and 4 different variants of PB2 suggests that reassortment occurs frequently in HPAI viruses in West Java, Indonesia. Of interest, 1 variant of PB2 was highly similar to LPAI from nearby countries: Malaysia (H5N2), Korea (H7N7, H3N8), Japan (H1N1), and Mongolia (H7N1); viruses that until recently had not been detected in Indonesia (*31*). A similar PB2 and putative reassortants with other LPAI viruses were recently reported (*13*). These results suggest that many more LPAI viruses are likely to circulate in Indonesia but are not detected because active surveillance in wild birds or poultry is not performed. Also, diagnostic procedures that solely focus on the detection of H5N1 viruses may contribute to missing influenza viruses of other subtypes.

The presence of multiple reassortants of HPAI viruses should be an alert to the regional and international community to strengthen mitigation action plans to prevent the further reassortment and genetic drift of the viruses. Preventing virus transmission between poultry flocks, stringent biosecurity measure in (wild) bird markets, and keeping poultry separated from wild birds will help to prevent introduction, adaptation, and reassortment of LPAI viruses to a possibly novel zoonotic HPAI virus as currently observed in China and other countries (*18*,*34*,*35*).

Structured, active surveillance in combination with genetic and phylogenetic analysis are urgently needed to reveal these viruses’ mutations and potential zoonotic effects, as the viruses rapidly and continually evolve with frequent reassortment (*36*). Also, adequate interventions at live poultry markets, such as separate markets for different poultry types with higher biosecurity and restructuring of the poultry chain, are crucial to prevent further loss from novel reassortant HPAI H5N1 viruses (*29*,*37*,*38*).

Appendix 1 Phylogenetic trees of gene segments for reassortments among avian influenza A(H5N1) viruses circulating in Indonesia, 2015–2016.

Appendix 2 Additional information about reassortments among avian influenza A(H5N1) viruses circulating in Indonesia, 2015–2016.
