# Supplementary material for: Reassortments among Avian Influenza A(H5N1) Viruses Circulating in Indonesia, 2015–2016
Source: Emerg Infect Dis. 2019 Mar;25(3):465–72. doi: 10.3201/eid2503.180167 (PMC6390736; doi:10.3201/eid2503.180167)
Supplement: Appendix 1 — Phylogenetic trees of gene segments for reassortments among avian influenza A(H5N1) viruses circulating in Indonesia, 2015–2016. [file 18-0167-Techapp-s1.pdf]

Article DOI: https://doi.org/10.3201/eid2503.180167

Reassortments Among Avian Influenza A(H5N1) Viruses Circulating in Indonesia, 2015–2016

Appendix 1

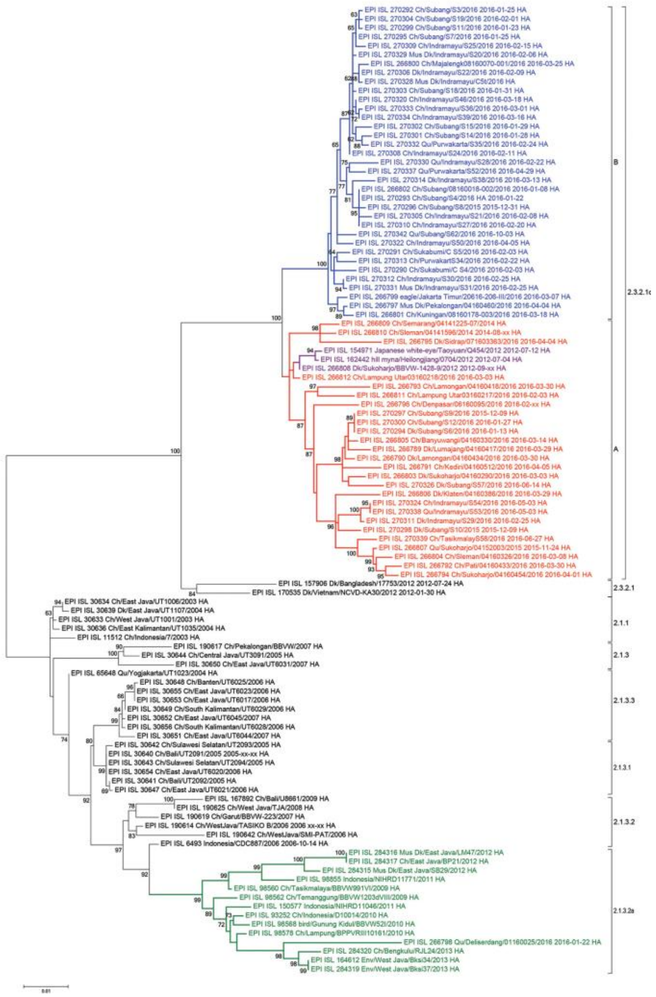

Appendix 1 Figure 1. Phylogenetic trees of HA segment of influenza A(H5N1) virus. Evolutionary history was inferred using the maximum-likelihood method based on the GTR+G model (*1*). The tree with the highest log likelihood is shown. The percentage of replicate trees in which the associated taxa clustered together in the bootstrap test (1,000 replicates) is shown next to the nodes. Scale bar represents number of substitutions per site. Evolutionary analyses were conducted in MEGA6 (*2*). Blue indicates subgroup B; red, subgroup A; purple, strains of clade 2.3.2.1c; green, clade 2.1.3.2a H5N1.

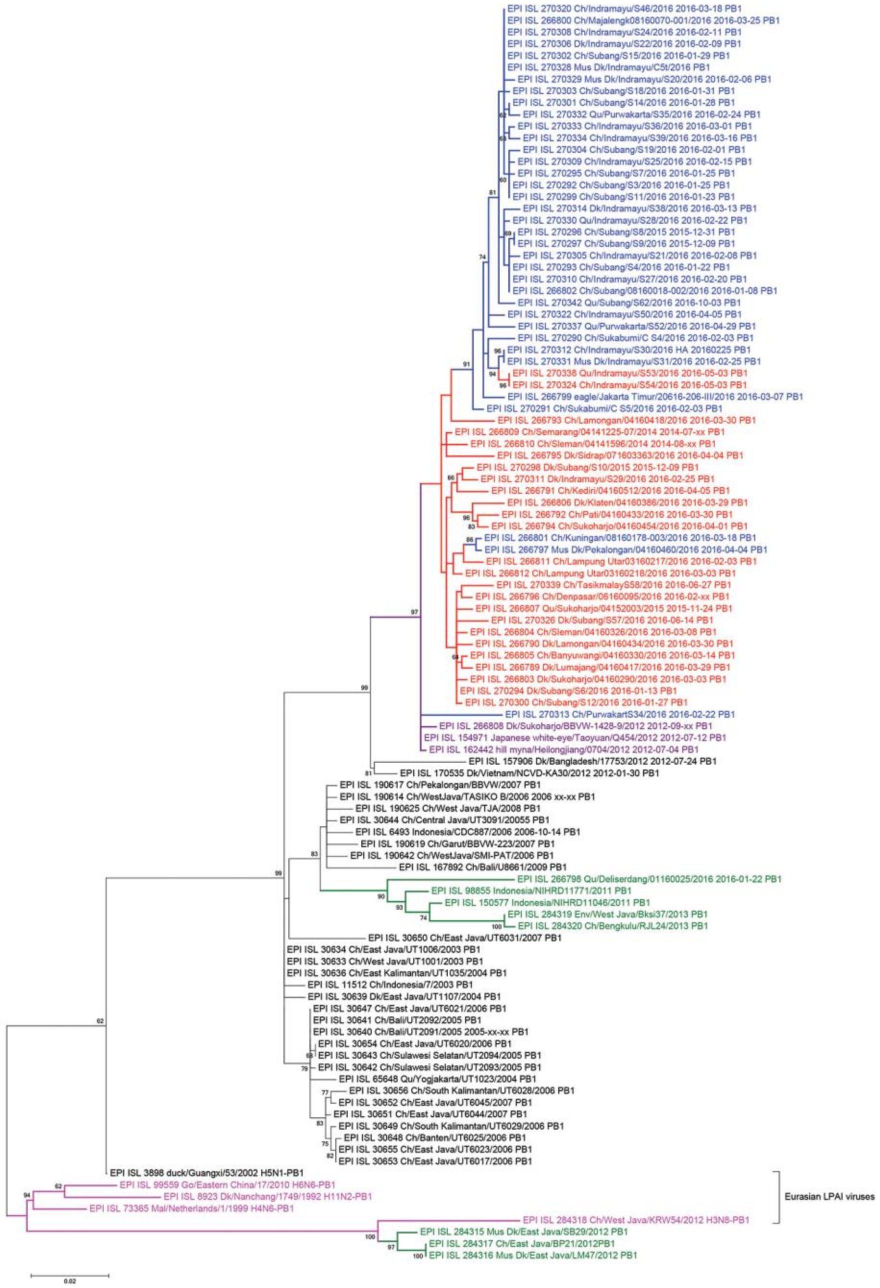

Appendix 1 Figure 2. Phylogenetic trees of PB1 segment of influenza A(H5N1) virus. Evolutionary history was inferred using the maximum-likelihood method based on the GTR+G+I model (1). The tree with the highest log likelihood is shown. The percentage of replicate trees in which the associated taxa clustered together in the bootstrap test (1,000 replicates) is shown next to the nodes. Scale bar represents number of substitutions per site. Evolutionary analyses were conducted in MEGA6 (*2*). Blue indicates subgroup B; red, subgroup A; purple, strains of clade 2.3.2.1c; green, clade 2.1.3.2a H5N1; pink, Eurasian LPAI viruses.

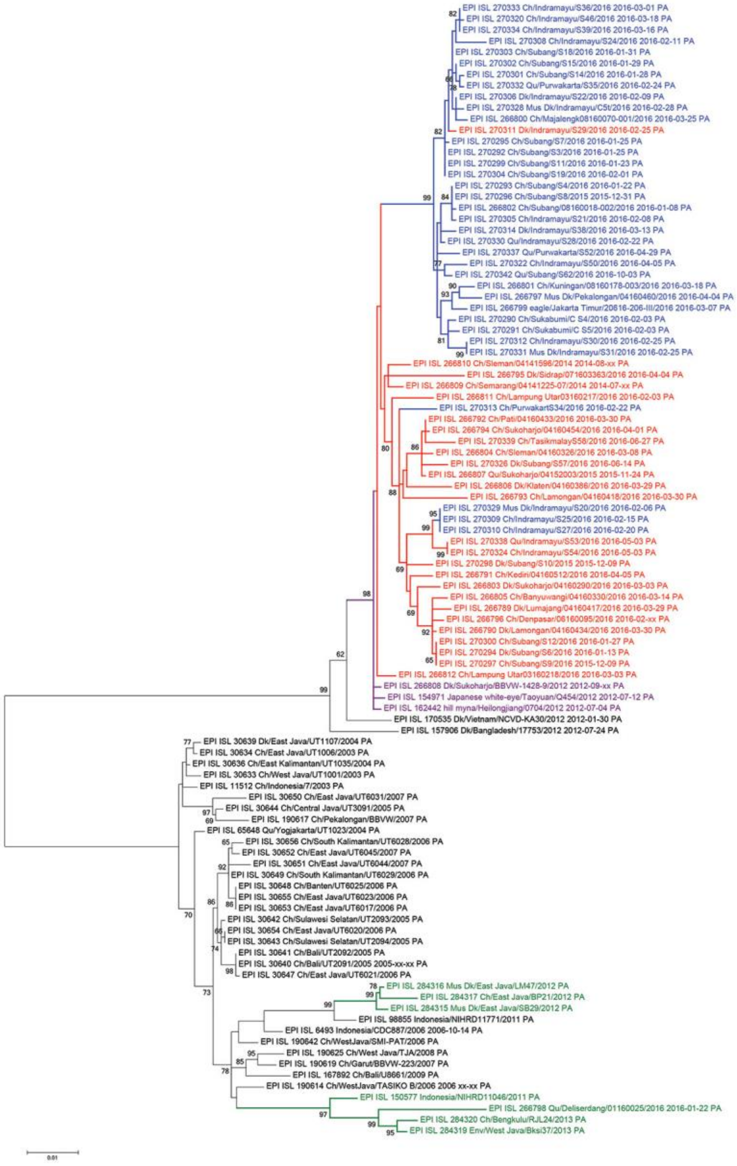

Appendix 1Figure 3. Phylogenetic trees of PA segment of influenza A(H5N1) virus. Evolutionary history was inferred using the maximum-likelihood method based on the GTR+G+I model (1). The tree with the highest log likelihood is shown. The percentage of replicate trees in which the associated taxa clustered together in the bootstrap test (1,000 replicates) is shown next to the nodes. Scale bar represents number of substitutions per site. Evolutionary analyses were conducted in MEGA6 (*2*). Blue indicates subgroup B; red, subgroup A; purple, strains of clade 2.3.2.1c; green, clade 2.1.3.2a H5N1.

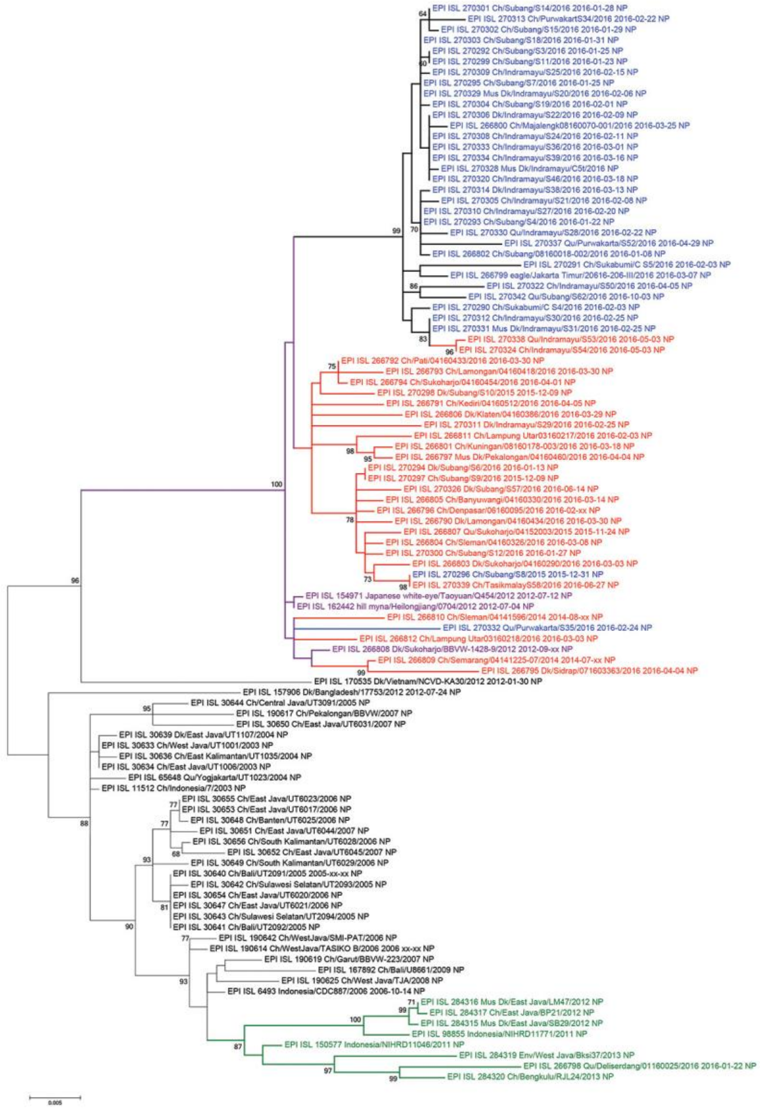

Appendix 1 Figure 4. Phylogenetic trees of NP segment of influenza A(H5N1) virus. Evolutionary history was inferred using the maximum-likelihood method based on the GTR+G+I model (1). The tree with the highest log likelihood is shown. The percentage of replicate trees in which the associated taxa clustered together in the bootstrap test (1,000 replicates) is shown next to the nodes. Scale bar represents number of substitutions per site. Evolutionary analyses were conducted in MEGA6 (*2*). Blue indicates subgroup B; red, subgroup A; purple, strains of clade 2.3.2.1c; green, clade 2.1.3.2a H5N1.

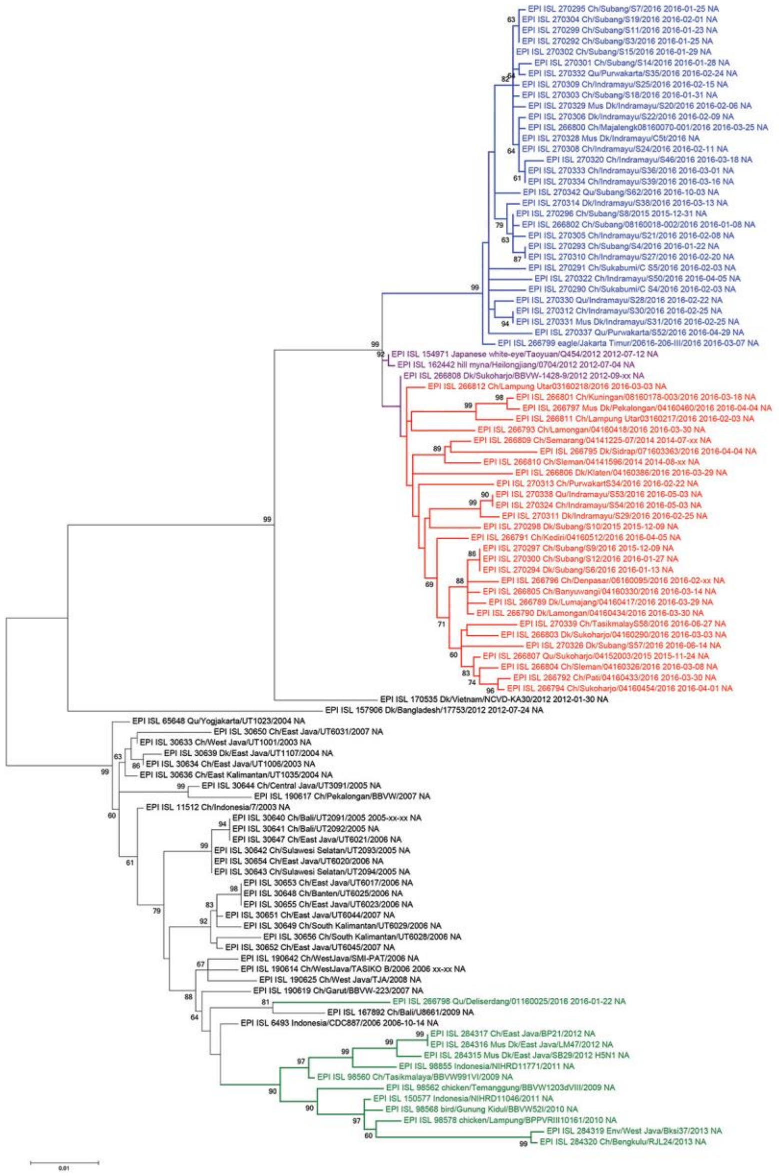

Appendix 1 Figure 5. Phylogenetic trees of NA segment of influenza A(H5N1) virus. Evolutionary history was inferred using the maximum-likelihood method based on the GTR+G+I model (1). The tree with the highest log likelihood is shown. The percentage of replicate trees in which the associated taxa clustered together in the bootstrap test (1,000 replicates) is shown next to the nodes. Scale bar represents number of substitutions per site. Evolutionary analyses were conducted in MEGA6 (*2*). Blue indicates subgroup B; red, subgroup A; purple, strains of clade 2.3.2.1c; green, clade 2.1.3.2a H5N1.

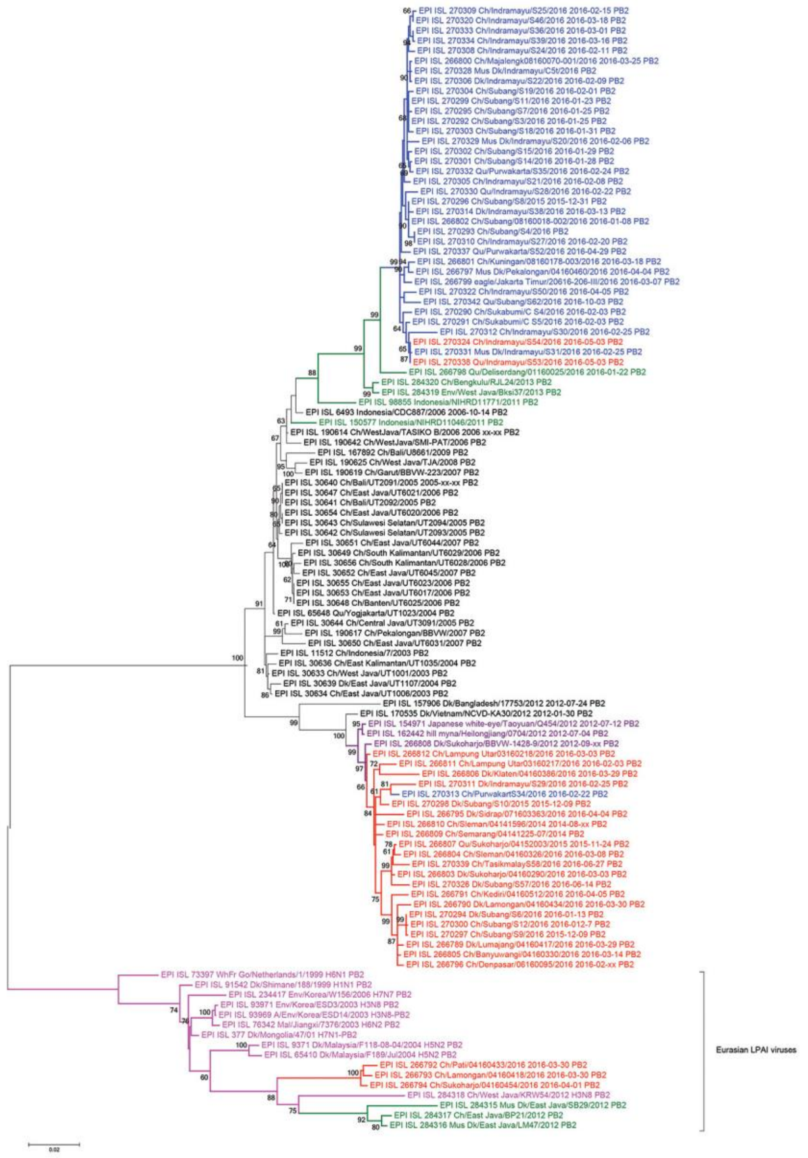

Appendix 1 Figure 6. Phylogenetic trees of PB2 segment of influenza A(H5N1) virus. Evolutionary history was inferred using the maximum-likelihood method based on the GTR+G+I model (1). The tree with the highest log likelihood is shown. The percentage of replicate trees in which the associated taxa clustered together in the bootstrap test (1,000 replicates) is shown next to the nodes. Scale bar represents number of substitutions per site. Evolutionary analyses were conducted in MEGA6 (*2*). Blue indicates subgroup B; red, subgroup A; purple, strains of clade 2.3.2.1c; green, clade 2.1.3.2a H5N1; pink, Eurasian LPAI viruses.

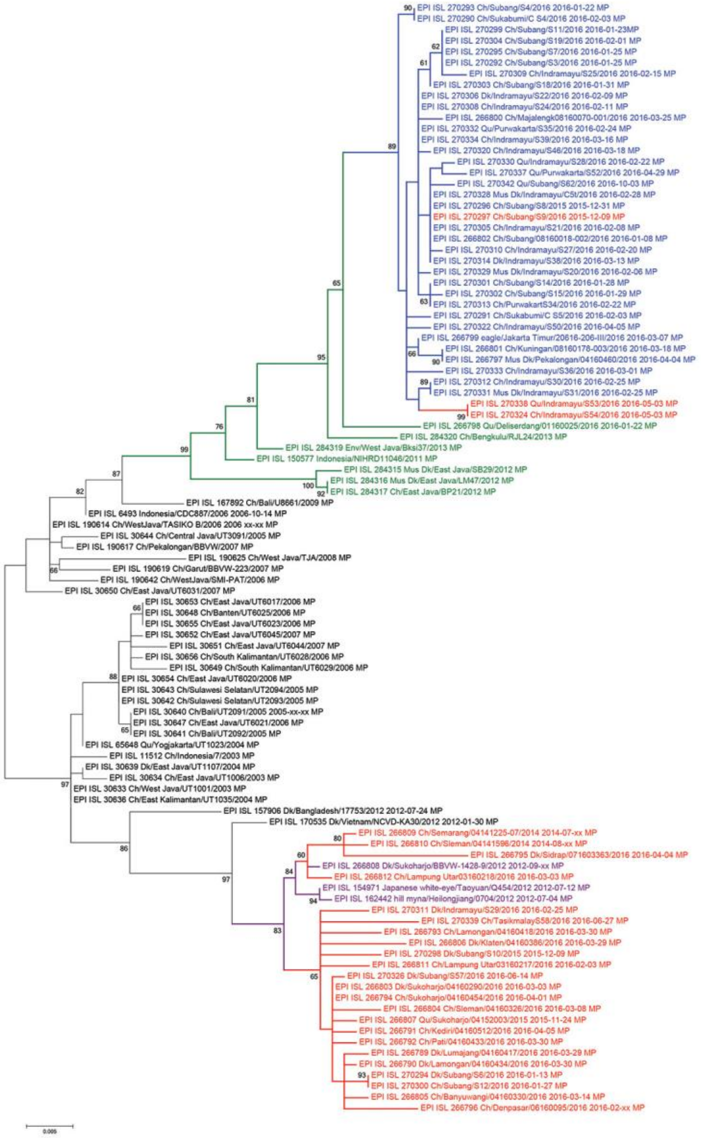

Appendix 1 Figure 7. Phylogenetic trees of MP segment of influenza A(H5N1) virus. Evolutionary history was inferred using the maximum-likelihood method based on the GTR+G+I model (1). The tree with the highest log likelihood is shown. The percentage of replicate trees in which the associated taxa clustered together in the bootstrap test (1,000 replicates) is shown next to the nodes. Scale bar represents number of substitutions per site. Evolutionary analyses were conducted in MEGA6 (*2*). Blue indicates subgroup B; red, subgroup A; purple, strains of clade 2.3.2.1c; green, clade 2.1.3.2a H5N1.

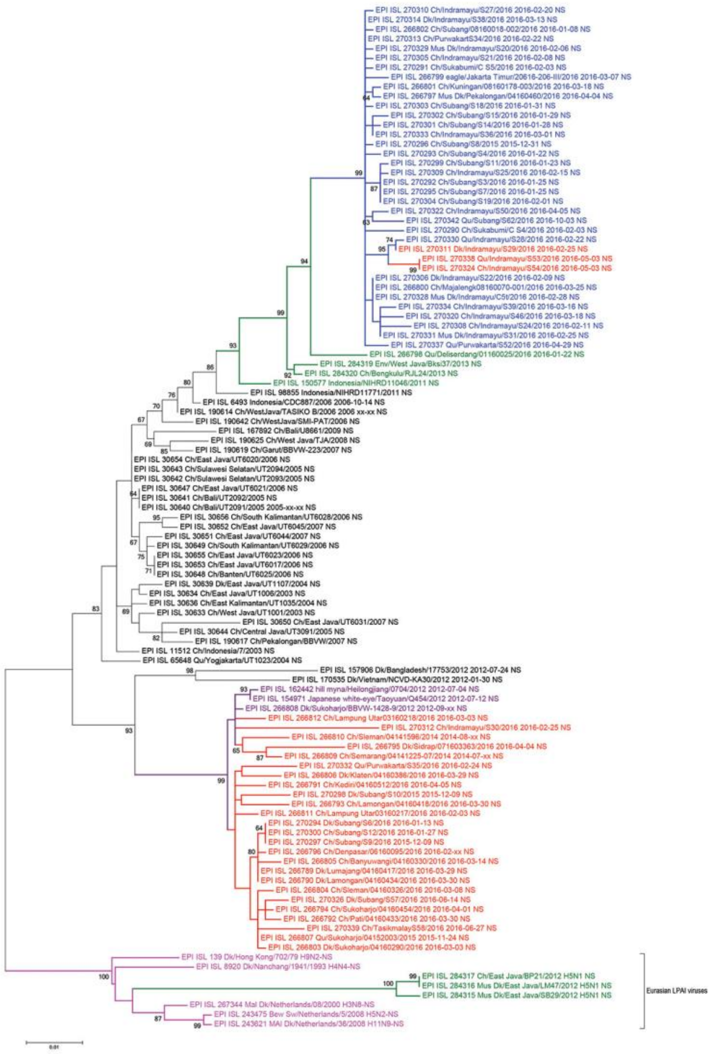

Appendix 1 Figure 8. Phylogenetic trees of NS segment of influenza A(H5N1) virus. Evolutionary history was inferred using the maximum-likelihood method based on the GTR+G+I model (1). The tree with the highest log likelihood is shown. The percentage of replicate trees in which the associated taxa clustered together in the bootstrap test (1,000 replicates) is shown next to the nodes. Scale bar represents number of substitutions per site. Evolutionary analyses were conducted in MEGA6 (*2*). Blue indicates subgroup B; red, subgroup A; purple, strains of clade 2.3.2.1c; green, clade 2.1.3.2a H5N1; pink, Eurasian LPAI viruses.

References

1. Nei M, Kumar S. Molecular evolution and phylogenetics. 2000. New York: Oxford University Press.

2. Tamura K, Stecher G, Peterson D, Filipski A, Kumar S. MEGA6: Molecular Evolutionary Genetics Analysis version 6.0. Mol Biol Evol. 2013;30:2725–9. PubMed http://dx.doi.org/10.1093/molbev/mst197
